# Cough syrup buyers get referred but not tested: private pharmacy–based tuberculosis surveillance pilot in Jaipur, India

**DOI:** 10.5588/pha.26.0017

**Published:** 2026-08-24

**Authors:** K. Gupta, A. Phatak, P. Manickam, V.N. Baig, H. Abdullah, Y. Singh, S. Jain, S. Nag, Y. Saxena, A.K. Bhardwaj, K. Roopchandani, D.K. Taneja, J. Meena, H.D. Shewade

**Affiliations:** 1 Department of Community Medicine, Geetanjali Institute of Medical Sciences, Jaipur, Rajasthan, India;; 2 State Drug Control Department, Swasthya Bhawan, Directorate of Medical & Health Services, Rajasthan, India;; 3 Division of Health Systems Research, ICMR National Institute of Epidemiology (ICMR-NIE), Chennai, Tamil Nadu;; 4 Department of Community Medicine, Rajasthan University of Health Sciences College of Medical Sciences, Jaipur, Rajasthan, India;; 5 Khushi Baby, Jaipur, India;; 6 National TB Task Force, New Delhi, India.

**Keywords:** tuberculosis, chemist, sputum testing, anti-tussive, TB case finding, MPHISOR

## Abstract

**SETTING:**

In the private sector of Rajasthan (north Indian state), missed tuberculosis (TB) notifications were seven times the reported notifications. The higher the private cough syrup sales in districts, the higher the missed TB notification.

**OBJECTIVE:**

We assessed the operational feasibility of engaging private pharmacies to refer cough syrup buyers for TB testing by the routine health system in Jaipur II district, Rajasthan.

**METHODS:**

This was a longitudinal descriptive study using aggregate secondary data. In collaboration with the state TB and drug control authorities, private pharmacy–based TB surveillance (PPTS) was piloted from November 2025 to January 2026. Random cross-verification of the care cascade data was done by designated district officials by telephonically contacting cough syrup buyers.

**RESULTS:**

Of 2,413 cough syrup buyers shared by pharmacies, 759 (31%) were reported as tracked and 334 (44%) of them underwent sputum examinations, yielding three (1%) PPTS-detected TB. After cross-verification, we estimated that against ≈2200 (90% of 2,413) private cough syrup buyers, there were an estimated ≈110 (30% of 334) PPTS sputum examinations and three PPTS-detected TB (3%).

**CONCLUSION:**

Private pharmacies have the potential to share cough syrup buyers’ details that can contribute to significant TB notifications. Tracking cough syrup buyers needs urgent attention.

Globally, the estimated annual tuberculosis (TB) incidence is 131 per 100,000 population.^1,2^ India constitutes 25% of the global TB burden, with an estimated annual incidence of 187 per 100,000.^3,4^ In 2024, India notified 2.5 million people with TB (PwTB) with an estimated 0.2 million missing TB.^1^ At least half of the people with TB symptoms in India seek care from the private sector.^5–7^ Following the efforts of India’s national TB elimination programme (NTEP) in engaging private sector, the annual TB notification rate has been on a rising trend during 2021–23, from 154 to 179 per 100,000 population.^3,4^ Rajasthan is one of the five states in India to account for half of the national TB burden.^4,8^ While the estimated prevalence of TB (2019–21) is 484 per 100,000, the state notifies 204 people per 100,000 annually (51 by private sector).^4^ This indicates a high prevalence to notification ratio (2.3:1) when compared to the national estimate (1.8:1). This gap calls for alternative strategies for case finding to supplement what is routinely implemented.

Cough as a frequent and prominent symptom of pulmonary TB is used as a part of identification of presumptive TB.^9–11^ An ecological study in Rajasthan showed that missed private TB notification (total persons taking treatment in private sector estimated using private drug sales minus private sector notifications) was seven times the reported private sector notification.^12^ District-level quarterly private cough syrup sales positively correlated with district-level missed private sector notification, district-level quarterly private sector notification and overall notification. One of the recommendations was routine surveillance of quarterly private cough syrup sales per district per 100,000 population and prioritising private sector engagement in districts with relatively higher private cough syrup sales.^12^ In addition to engaging private health care providers, private sector engagement may also include private pharmacy–based TB surveillance (PPTS) involving tracking of people approaching private pharmacies for buying cough syrups.^12^ Pharmacy medical sales have been used during COVID-19 for syndromic surveillance and the same could be a used for TB surveillance.^13^ In Patna, Bihar, during 2015–2017, private pharmacies contributed towards improved TB detection and notification.^14^ Their referral intervention was implemented via recruitment of pharmacies engaged in an externally funded public–private mix programme.^14^ Involving pharmacies within the routine health system setting utilising existing workforce is the need of the hour.

Following our ecological study from Rajasthan,^12^ the state TB and drug control authorities designed a PPTS pilot in Jaipur II district to track people buying cough syrups and get them or their household members tested for TB if they fell under the presumptive TB criteria. Specific objectives were to assess the feasibility of involving private pharmacies within the routine health system setting to refer cough syrup buyers for TB testing.

## METHODS

We used a longitudinal descriptive design using routinely collected aggregate secondary data. Longitudinal descriptive design was required since data were collected from routine health system for three consecutive months in order to find out the proportion of cough syrup buyers who tested positive for TB.

### General setting

At the country level, the NTEP is led by the Central TB Division under the Ministry of Health and Family Welfare.^3^ The state of Rajasthan (largest state by area, arid to semiarid, 80 million population) in north-west India presents unique challenges and opportunities for addressing TB due to its diverse socio-economic landscape and varying health care access. It has 42 administrative districts as of May 2025. The state and the district TB cells govern the activities of the programme at the state and district level, respectively. At the sub-district level, activities are organised under the TB unit.^7^

### Specific setting

Jaipur district was chosen as it has one of the highest private cough syrup sales per 100,000 population per district in Rajasthan.^12^ Under the NTEP, Jaipur is further divided into two administrative districts. Jaipur II has a population of 3.2 million with 16 TB units (5 urban and 11 rural) and 104 TB diagnosis centres (TDCs). In rural TB units, the block medical officers handle both NTEP activities and other health-related activities. In urban TB units, there is a separate NTEP medical officer along with a block medical officer. During the study period, but for the three rural TB units that were supported by a single TB senior treatment supervisor (STS), all other TB units had one STS per TB unit.

### Study population

All residents of Jaipur II, who visited the private pharmacies of Jaipur II to purchase cough syrups between November 2025 and January 2026 and consented to provide their contact details to the pharmacies were included (irrespective of whether they fitted NTEP’s presumptive TB criteria or not). There were ≈2,500 private pharmacies in Jaipur II.

### Private pharmacy–based TB surveillance pilot

PPTS was implemented as a 3-month pilot in routine health system using available workforce. At the district level, a repository of WhatsApp numbers of private pharmacies, public TDCs, STS, and accredited social health activists (ASHAs – women health volunteers at village/ward level) were prepared. Two district-level nodal persons (DNPs) were identified, district TB officer of Jaipur II and KG (primary author). Their WhatsApp numbers were identified and mentioned in the Government orders. A PPTS standard operating procedure that was circulated by the state TB officers guided training, implementation, and monitoring.

After seeking verbal consent, the cough syrup buyer details (name, address, phone number, and date of purchase) were collected by the pharmacies and shared with the DNPs via an online form. Every week, this list of cough syrup buyers was shared with the medical officer of the TB unit and TB STS and through them to the ASHAs. In rural TB units, cough syrup buyers’ data had to be shared with the block medical officer to get them tracked by ASHAs. Whereas in urban Jaipur, the cough syrup buyers’ data had to be shared with the NTEP medical officer and then this officer shared it with the block medical officer to get the buyers’ tracked by ASHAs. The ASHA made home visit and confirmed the persons in the household for whom cough syrup was purchased and collected his/her sputum if the person fulfilled presumptive TB case criteria. ASHAs were incentivised for every sputum collection/transport done and TB diagnosis as per existing guidelines.

In the TDC where sputum examination happened, the laboratory technician mentioned ‘PPTS’ against the entry in the laboratory register (presumptive sputum examination register). In a locally stored MS Excel file, based on updates by the STS, the DNPs maintained the line list of people purchasing cough syrup and tracked their status (name of referring private pharmacy, the name of TDC to which the person belongs to, home visit, sputum collection transport and examination, result of examination).

**FIGURE 1.**
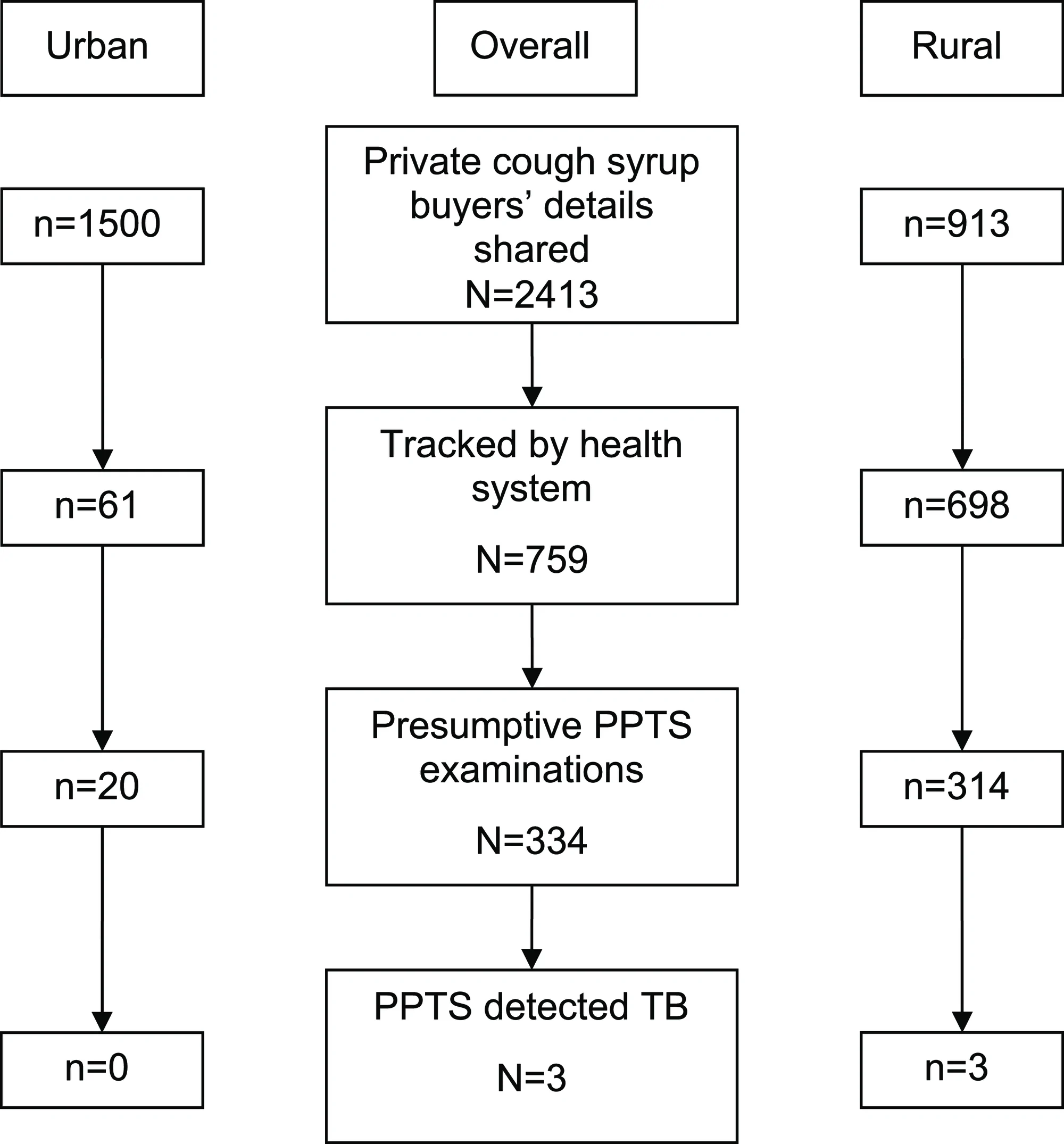
Private pharmacy–based tuberculosis (TB) surveillance (PPTS) pilot care cascade, stratified by urban and rural TB units, Jaipur II district, India, November 2025 to January 2026.

Random cross-verification of aggregate numbers in the care cascade was done through phone calls to the cough syrup buyers by the DNPs within a month: at least 50% of the cough syrup buyers, 20% of the presumptive TB examined, and 100% of the PPTS-detected TB. The person detected with TB were notified and started on anti-TB treatment (Figure 1). By the 15th of next month, for each monthly cohort of private cough syrup buyers (based on date of purchase), PPTS monitoring indicator slide was reviewed in the monthly review meetings conducted by the district TB cell (see https://doi.org/10.6084/m9.figshare.31744963).

### Variables, sources of data, and data analysis

MS Excel sheet maintained by the DNPs at the district level were used to generate aggregate numbers (overall and monthly) of cough syrup buyers, of them numbers (proportion) undergoing sputum examination, and of them the test positivity. These results along with findings of cross-verification were presented as frequency and percentages. Subject to favourable results of cross-verification, if at least 50% of the cough syrup buyers (or their household members) were tested with a test positivity of at least 3%–5%, we would consider this to be a feasible strategy to scale up in all districts.

### Ethical statement

Ethics approval was obtained from the Institutional Human Ethics Committee of ICMR National Institute of Epidemiology (ICMR-NIE), Chennai, India (NIE/IHEC/A/202506-22 dated 30 June 2025). As this operational research involved the use of aggregate secondary data without the use of identifiers, a waiver for written informed consent was sought and approved by the ethics committee. Administrative approval was obtained from the Director Public Health of Rajasthan, State TB Office, and State Drug Controller, Rajasthan.

## RESULTS

Of the 2,413 cough syrup buyers reported, 913 (38%) were from rural and 1,500 (62%) were from urban TB units. Eleven TB units (nine rural and two urban) shared 78% cough syrup buyers. Of the 2,413, month-wise distribution was 985 in November 2025, 943 in December 2025, and 485 in January 2026. Of the 2413, a total of 759 (31%) were reported as tracked, and of them 334 (44%) were reported as presumptive TB and examined for TB at the TDC. Of the 334, three (1%) tested positive for TB (see Table). The reported PPTS care cascade stratified by urban and rural TB units is depicted in Figure 1. Though 62% of cough syrup buyers were from urban TB units, 92% of those tracked, 94% PPTS examinations, and all PPTS-detected TB were from rural TB units.

**TABLE.** Monitoring indicators for the pilot of private pharmacy–based tuberculosis (TB) surveillance (PPTS) in Jaipur II district, India, November 2025 to January 2026.

Monitoring indicators	Overall		Nov 2025		Dec 2025		Jan 2026	
	N	%	N	%	N	%	N	%
Cough syrup buyers who consented and provided details	2,413^A^		985		943		485	
Reported sputum examinations among cough syrup buyers	334^B^	13.8	76	7.7	129	13.6	129	26.5
Reported PPTS-detected TB among examinations	3^C^	0.8	1	1.3	2	1.5	0	0

Results of cross-verification of reported PPTS care cascade data.

A 90% fulfilled study participant criteria.

B 30% were truly examined.

C All were confirmed to be PPTS-detected and initiated on treatment.

**FIGURE 2.**
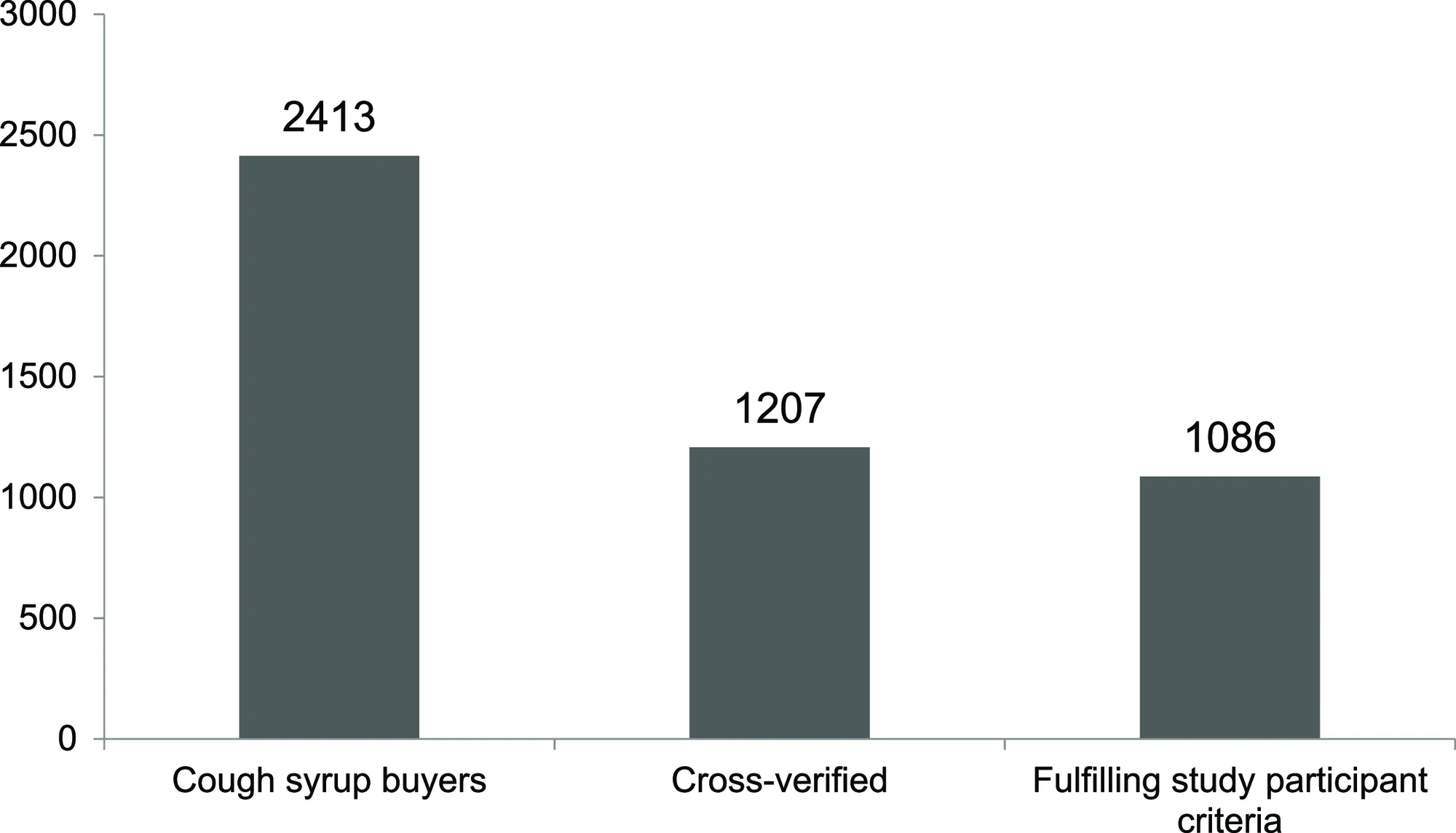
Cross-verification indicators for cough syrup buyers for the pilot of private pharmacy–based tuberculosis (TB) surveillance in Jaipur II district, India, November 2025 to January 2026.

**FIGURE 3.**
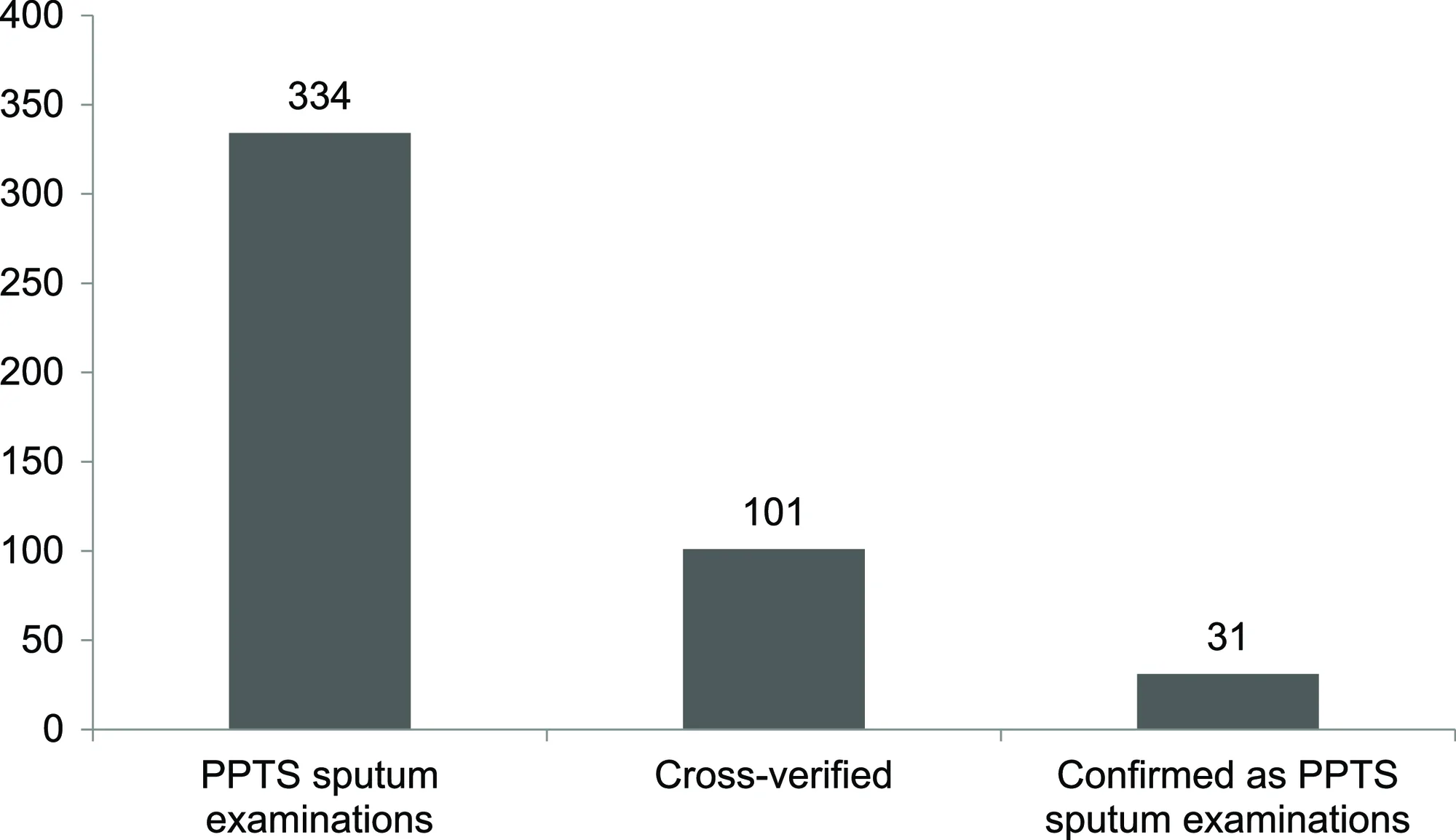
Cross-verification of presumptive tuberculosis (TB) cases for the pilot of private pharmacy–based TB surveillance (PPTS) in Jaipur II district, India, November 2025 to January 2026.

We met the cross-verification targets that were set. Of the 2,413 cough syrup buyers, 1,207 (50%) were randomly cross-verified and of them 1,086 (90%) met the study population criteria (resided in Jaipur II and purchased cough syrup from a pharmacy in Jaipur II). Of the 334 reported PPTS presumptive examinations, 101 (30%) were randomly cross-verified, and of them 31 (30%) were confirmed as PPTS-detected presumptive TB. All three reported PPTS-detected TB were confirmed to be detected through PPTS (Figures 2 and 3).

After considering our cross-verification findings, we estimated that against ≈2,200 (90% of 2,413) private cough syrup buyers, an estimated ≈110 (30% of 334) PPTS sputum examinations and three PPTS-detected TB (3%) were identified.

## DISCUSSION

Building on an ecological study on higher correlation between private cough syrup sales and TB notification rates in Rajasthan state, we assessed operational feasibility of using a network of private pharmacies in Jaipur district to surveil, refer, and test cough syrup buyers for TB during a 3-months period. In this pilot engagement of pharmacies within the routine health system setting, tracking of the buyers by the system was sub-optimal and upon cross-verification, pharmacies were found to be sharing more reliable data than the TB units.

The strengths of our study included implementation in routine operational settings by the existing workforce. Therefore, the findings were grounded in reality. PPTS was co-designed by the State Drug Control Department and State TB Office indicating ownership, thereby increasing the chances of adopting the learnings from this pilot. Cross-verification of the cough syrup buyers–reported PPTS examinations and PPTS-detected TB helped generate reliable information. As operational research was built within existing systems, we met with a few limitations. Documentation errors typical of routine data cannot be ruled out. The operational research was based on aggregate data and the reasons for the gap between tracked and examined cough syrup buyers were not systematically captured. Limitations notwithstanding, we discuss below the two key programme relevant findings. First, PPTS did not meet our feasibility assessment criteria. Based on the findings of cross-verification, only 5% (110/2200) of cough syrup buyers underwent sputum examination with a test positivity of 3%. The key reason for this was that cough syrup buyers were not tracked. This could be speculated due to lack of staff against the norms, overworked NTEP staff, and not examining using sputum microscopy in the absence of chips and cartridges for rapid molecular diagnostic tests. Among those reported to be tracked, due to time constraints, TB unit staff shared low-quality data that led to poor results in cross-verification. The performance in tracking cough syrup buyers was relatively better in rural TB units than urban. This reflects existing coordination mechanisms in place affecting routine NTEP activities, not just PPTS. In rural areas, as there is no separate medical officer for NTEP, the district TB officer works directly with the sub-district level medical officer. ASHAs take instruction from block medical officers. Though cough syrup buyers’ details were shared (≈800 per month), tracking of the cough syrup buyers by the health system was sub-optimal. There is further scope for improvement for sharing cough syrup buyers: 2,500 pharmacies shared details of 2,413 cough syrup buyers over 3 months (one cough syrup buyer per pharmacy over 3 months). There appears to be a greater potential demand for tracking cough syrup buyers than reported in this pilot. This strategy has the potential to increase presumptive examinations subject to the health system’s capacity to test them. If ≈800 private cough syrup buyers per month increases to ≈2,400 and half of them (≈1,200) get examined with a test positivity of 3% (as estimated in our pilot following cross-verification), we can expect 36 TB notifications per month. Considering ≈75 public TB notifications per month in Jaipur II, this is a significant number.^4^ Second, cross-verification revealed that pharmacies shared more reliable data when compared to the TB units and helped us generate the potential of PPTS and infer the test-positivity more reliably. Based on the above findings and that of results from national-level TB active case-finding evaluations,^15^ and our programme experience, we recommend that any systematic case-finding initiative other than passive case finding should be backed by a certain percentage of aggregate numbers cross-verified across the care cascade.

In a similar study by Sharma et al.^16^ in Central Delhi, India, 871 new TB cases were identified in 10 months contributing to increase in TB case notification from 271 to 871 per 100,000 population. In Pakistan, a community pharmacy–referral network was established for 1 year in 2017. Every fifth referral among presumptive counselled at pharmacies was diagnosed with TB.^17^ A major limitation was that no cross-verification was done to confirm that reported TB detection happened truly through pharmacies. When the pharmacies shared cough syrup buyers’ details in the online form, in some instances DNPs or STS could not identify the TB unit based on the address provided. This calls for a single-window platform (say a mobile application) that could be used by all stakeholders (pharmacies, DNPs, NTEP medical officers, block medical officers, STS, and ASHAs) for identification and tracking of cough syrup buyers. The mobile application may auto-derive the TB unit based on the postal code and address of the cough syrup buyer. The design and testing of this mobile application should be guided by operational research.

## CONCLUSIONS

Although private pharmacies have the potential to share many cough syrup buyers’ details that can potentially contribute to significant sputum examination and TB notifications, the health system in Jaipur II is not geared up to test them. Possible policy guidelines for the province can be that state TB department and state drug control department can work together and track the cough syrup buyers by local TB staff to determine if they fulfil the criteria of presumptive TB case. This strategy will improve the yield of TB testing. Future operational research in this area could consider developing a mobile application to identify, record, and track presumptive TB among cough syrup buyers. PPTS should be complemented with cross-verification mechanisms especially by external monitors. As this pilot was co-designed by state level departments, we are confident that the findings of this operational research will guide modifications to close the gaps, improve tracking, and ensure testing of cough syrup buyers identified through pharmacies.

## Supplementary Material
