# Supplementary material for: Cough syrup buyers get referred but not tested: private pharmacy–based tuberculosis surveillance pilot in Jaipur, India
Source: Public Health Action. 2026 Aug 24;16(3):145–9. doi: 10.5588/pha.26.0017 (PMC13502715; doi:10.5588/pha.26.0017)
Supplement: Supplementary file 1 [file pha26-0017_supplementarydata1.pdf]

<u>Supplementary material.</u> Monitoring slide for routine monthly review meeting sunder the pilot of private pharmacy-based TB surveillance (PPTS) in Jaipur II district, India, November 2025 – January 2026

Rajasthan PPTS initiative – Pilot in Jaipur II
Monitoring Indicators

District / TB unit name:
Period Range of monitoring (based on date of cough syrup purchase):
Date when aggregate numbers and indicators were generated:

**Monitoring indicator**	**n**	**(%)**
Number of cough syrup buyers who provided details - a	w	
Number of PPTS entries (presumptive TB examinations) – b	x	b/a * 100
Number of PPTS-detected TB notified – c	y	c/b * 100
Number of PPTS-detected TB notified and initiated on treatment - d	z	d/c * 100

To be generated at TB unit level and district level every week and every month/ quarter and to be reviewed at TB unit and district level during monthly and quarterly review meetings. Monthly/ quarterly indicators will be generated during second week of next month.

Rajasthan PPTS initiative – Pilot in Jaipur II
Quality Indicators

District / TB unit name:
Period Range of monitoring (based on date of cough syrup purchase):
Date when aggregate numbers and indicators were generated:

**Quality indicator**	**n**	**(%)**	**Target**
Number of PPTS entries (presumptive TB examinations)- a	w		
Number of PPTS entries (presumptive TB examinations) cross-checked – b	x	b/a * 100	20 %
Number of PPTS-detected TB notified – c	y		
Number of PPTS-detected TB notified cross-checked - d	z	d/c * 100	100%

To be generated at TB unit level and district level every week and every month/ quarter and to be reviewed at TB unit and district level during monthly and quarterly review meetings. Monthly/ quarterly indicators will be generated during second week of next month.
